# Commentary: The association between serum vitamin C levels and respiratory infections in children and adolescents

**DOI:** 10.3389/fnut.2025.1654402

**Published:** 2025-11-19

**Authors:** Mei Zhaojun, Long Yang, Xie Wanyu, Li Jingjing

**Affiliations:** 1 Luzhou Maternal and Child Health Hospital, Luzhou, China; 2 Luzhou Second People's Hospital, Luzhou, China; 3 Luzhou Women and Children's Hospital, Luzhou, China

**Keywords:** commentary, serum vitamin C, respiratory infections, adolescents, children

We read with great interest the study by Li et al. (1), which examined the association between serum vitamin C concentrations and respiratory infections (RIs) among children and adolescents using data from the 2017–2018 National Health and Nutrition Examination Survey (NHANES). This work provides timely insights into the nutritional determinants of pediatric respiratory health. The authors should be commended for applying rigorous statistical models and leveraging a nationally representative dataset to address an important public health issue.

While we concur with the main findings, we wish to expand on several conceptual and methodological dimensions to further contextualize their significance and refine the interpretive framework.

## 1 Differentiating serum vitamin C status from supplementation effects

The study's use of cross-sectional serum vitamin C levels provides valuable epidemiological evidence but must be interpreted distinctly from outcomes of supplementation trials. Randomized controlled trials (RCTs) have shown that regular vitamin C supplementation does not consistently prevent common colds in the general population but can shorten illness duration by approximately 14% in children and 8% in adults, particularly under conditions of physical stress such as athletic training or military service (2). These RCTs complement but do not directly equate to serum concentration studies, as circulating vitamin C reflects dietary intake, absorption, and metabolism rather than supplementation alone. Clarifying this distinction avoids overinterpretation of causality and situates Li et al.'s findings appropriately within the continuum from nutritional status to interventional efficacy.

## 2 Contextualizing related micronutrients and levels of certainty

Li et al. briefly reference the potential interactions between vitamin C and other micronutrients such as zinc and vitamin D. This multidimensional nutritional perspective is valuable, yet it warrants further nuance. Systematic reviews report that zinc supplementation modestly reduces the duration of common colds but yields inconsistent results depending on formulation and dose (3). Similarly, vitamin D exhibits a protective effect mainly in deficient individuals (4) but more recent evidence (5) suggests attenuated or null associations. Distinguishing between hypothesized and established effects will enhance interpretive precision and scientific balance. Moreover, synergistic micronutrient interactions—while biologically plausible—remain theoretical and require validation through well-controlled factorial trials.

## 3 Laboratory context and methodological precision

To strengthen reproducibility, the commentary should briefly note the NHANES laboratory method (isocratic high-performance liquid chromatography with electrochemical detection) and the commonly applied deficiency threshold of < 23 μmol/L for serum vitamin C. Including such technical detail would clarify measurement validity and improve comparability with other population-based studies. Additionally, the reliance on self-reported RIs introduces recall bias; combining questionnaire data with biomarker-confirmed infections (e.g., C-reactive protein or procalcitonin) in future analyses would yield more robust and clinically meaningful outcomes.

From a causal standpoint, the cross-sectional nature of NHANES precludes directional inference. Incorporating approaches such as Mendelian randomization—using genetic variants related to vitamin C transporters (e.g., SLC23A1, SLC23A2)—could reduce confounding and reverse causality (6). These methods would strengthen causal plausibility between vitamin C and infection risk.

## 4 Integrating observational and interventional evidence

A balanced interpretation benefits from linking population-level associations with mechanistic and interventional evidence. Vitamin C's immunomodulatory effects—through antioxidant activity, leukocyte function enhancement, and epithelial barrier maintenance—provide biological plausibility for its observed associations with RIs (7). Observational studies, such as Li et al.'s, identify population patterns, while RCTs clarify causality under controlled conditions. Integrating both lines of evidence supports a coherent narrative: vitamin C sufficiency contributes to immune resilience, but supplementation benefits may be context-dependent. Future longitudinal or hybrid designs could better capture temporal dynamics and physiological thresholds for protective effects.

## 5 Translational and policy implications

From a translational perspective, Li et al.'s results reinforce the public health importance of nutrition-sensitive interventions. However, dietary diversification and fruit–vegetable intake should remain the cornerstone strategies rather than indiscriminate supplementation. Global nutrition frameworks, including those by the WHO and FAO, advocate prioritizing food-based approaches and reserving supplementation for at-risk subgroups—such as children with food insecurity, chronic disease, or increased oxidative stress. Embedding nutritional screening into pediatric preventive care, particularly within school-based health programs, could help identify subclinical deficiencies and guide targeted interventions. Moreover, such strategies align with the Sustainable Development Goals (SDG 2 and 3), linking nutrition to child wellbeing and infection resilience.

## 6 Conclusion

In summary, Li et al. have made an important contribution to understanding micronutrient–infection interactions in pediatric populations. Their work underscores the role of vitamin C as a modifiable determinant of respiratory health. Yet, to translate these findings into effective clinical or policy interventions, future research should distinguish serum status from supplementation effects, integrate interventional data, clarify micronutrient synergies, and adopt advanced causal designs. Such refinements will move the field closer to evidence-based, equitable strategies for improving child immune health worldwide.
